# The mechanism of the ornamental plant variety rights value formation and enhancement strategy based on SEM-SD

**DOI:** 10.1371/journal.pone.0336934

**Published:** 2025-12-19

**Authors:** Yongxia Chen, Zhichen Zhang, Deqian Kong, Zhe Rao, Wenna Li

**Affiliations:** 1 College of Civil Engineering, Nanjing Forestry University, Nanjing, Jiangsu, China; 2 Department of Statistics, University of British Columbia, Vancouver, British Columbia, Canada; Zhejiang Agriculture and Forestry University: Zhejiang A and F University, CHINA

## Abstract

With the acceleration of global urbanization, ornamental plants are playing an increasingly critical role in urban greening and climate regulation. Beyond improving urban landscapes, they mitigate the urban heat island effect through transpiration and enhance overall environmental quality. In developed countries, the surge of home gardening during the pandemic greatly stimulated the ornamental plant market, heightening interest in new variety development and the commercialization of plant variety rights. However, many newly bred ornamental varieties have failed to be effectively translated into productive resources, or their value has been underestimated, thereby constraining marketization. This study investigates the value formation mechanism of the Ornamental Plant Variety Rights (OPVR) and proposes optimization pathways for value enhancement. An OPVR value evaluation index system was first constructed through systematic literature review, expert consultation, and value chain analysis. A structural equation model (SEM) was then applied to examine the interrelationships among influencing factors, followed by a system dynamics (SD) model to explore the dynamic evolution of OPVR value and factor sensitivities. The SEM results indicate that four categories of factors contribute to OPVR value formation in the following order: variety value (Var)> technological level (Tec)> market and brand (Mar)> intellectual property protection (IPP). Specifically, variety value exerts both direct and indirect effects through its positive influence on technology and market performance, while IPP, though less influential, positively reinforces variety and market factors. The SD simulations further reveal that factor impacts are comparable during the first four years but diverge thereafter, with sensitivity ranking as follows: market and brand > technological level > variety value > intellectual property protection. Overall, this study clarifies the mechanisms underlying OPVR value formation and offers actionable insights for enterprises to design evidence-based strategies that enhance variety value, strengthen market positioning, and improve long-term competitiveness.

## 1 Introduction

The growing demand for urban greening, coupled with the rapid rise of home gardening, has rendered traditional ornamental plant varieties insufficient to meet the evolving market expectations for high-quality and diverse plants. This transformation underscores the urgent need to develop new ornamental plant varieties in response to the requirements of modern urban greening. Studies have shown that the COVID-19 pandemic acted as a catalyst in significantly promoting the trend of home gardening, which in turn spurred rapid growth in the demand for new ornamental plant varieties [1]. This trend suggests that as household demand for personalized and high-quality ornamental plants continues to increase, the acceptance and commercialization potential of new varieties have risen markedly. However, in the absence of robust plant variety rights (PVRs) protection and effective value realization mechanisms, these potential market demands are difficult to translate into tangible industrial value. Against this backdrop, the effective protection of Ornamental Plant Variety Rights (OPVR) is not only crucial for safeguarding breeders’ interests but also directly affects the commercialization process of new varieties and the competitiveness of seed enterprises. Nevertheless, the development of the ornamental plant industry remains severely constrained by infringement issues. On the one hand, there are malicious infringements such as unauthorized propagation and sales; on the other hand, unintentional infringements are widespread due to insufficient awareness among the public and some producers regarding the protection status of variety rights [2]. Combined with relatively weak legal systems and limited enforcement in ornamental plant protection, infringements occur frequently. Such an environment not only undermines the effectiveness of PVRs but also increases uncertainty in the research, development, and promotion of new varieties. In the Chinese context, the coexistence of commercialization difficulties and infringement issues has led to an undervaluation of OPVR, further diminishing breeders incentives for innovation [3].

Although existing studies have explored plant variety rights from multiple perspectives, significant limitations remain. First, Di Fonzo’s research has focused primarily on food and cash crops, leaving systematic studies on ornamental plant variety rights notably insufficient [4,5]. Second, most of the existing literature concentrates on legal protection and institutional arrangements, lacking in-depth analyses of the unique value attributes of OPVR, such as aesthetic, cultural, and brand values [6–8]. Third, in terms of research methods, although some scholars have pointed out that system dynamics (SD) is suitable for revealing the dynamic mechanisms of value chains [9], and that structural equation modeling (SEM) is effective in testing complex causal relationships [10], no study to date has combined both approaches to investigate the value formation mechanisms of OPVR.

Therefore, this study aims to fill these research gaps. Specifically, (1) to comprehensively identify the key factors influencing the value of ornamental plant variety rights based on a systematic literature review and expert consultation, and to construct a value chain framework; (2) to employ survey data and SEM to investigate the mechanisms of OPVR value formation and reveal the relative roles of various factors; (3) to integrate system dynamics to simulate the evolution of OPVR value over the next decade and identify the critical drivers of value enhancement; and (4) to propose targeted strategies for value improvement, thereby advancing the commercialization and industrialization of ornamental plant variety rights.

## 2 Literature review

### 2.1 Value chain theory

Value chain theory, proposed by Porter in 1985, explains how firms generate and deliver value through interrelated activities in production and operations [11]. Its core premise is that labor division and collaboration not only drive internal value creation but also enhance competitiveness through interaction among links [12]. The framework has since expanded to industrial, global, knowledge, and innovation value chains [13–16]. In agriculture, it has been widely applied to crops such as grain, cotton, coffee, and fruits, tracing value creation and distribution from breeding to sales [17–19]. These applications show that value chain analysis can clarify how organization and coordination enhance competitiveness. More recently, the framework has been applied to digital agriculture and AI-driven transformation [20]. Overall, it is a vital tool for analyzing value creation, transmission, and realization in complex industries. However, its systematic application to ornamental plant variety rights remains limited, and applying it here may help clarify value creation across different stages and provide a theoretical foundation for value enhancement pathways.

### 2.2 Plant variety rights

Plant variety protection systems have fostered favorable legal environments for enterprises and research institutions, promoting breeding innovation and global competitiveness [21]. Since the adoption of UPOV in 1961, a unified legal framework has stimulated innovation, standardized practices, and strengthened competitiveness [22].

Research on PVRs has mainly taken two perspectives. First, institutional and legal studies conclude that PVRs stimulate innovation, clarify property boundaries, and promote standardization [17,23]. At the international level, the 1978 and 1991 UPOV versions serve as benchmarks for analyzing institutional differences and policy choices [8]. Yet in developing economies, weak enforcement and high legal costs reduce incentives [24]. Second, economic and industrial research shows that PVR protection can increase R&D, promote varietal improvement, and enhance competitiveness [7]. Analyses of application, authorization, licensing, and transfer reveal persistent challenges: limited commercialization, underdeveloped value assessment, and regional disparities. Such gaps between institutional frameworks and market transformation constrain full commercial potential [25].

In sum, existing studies provide a theoretical foundation but remain limited in two respects: (1) focus is overly concentrated on legal and policy dimensions with insufficient empirical exploration of industrialization and markets; and (2) systematic studies on how OPVR value is formed, transmitted, and enhanced within the value chain are lacking.

### 2.3 Value enhancement of plant variety rights

With the increasing use of value chain theory in agricultural research, scholars have extended it to PVRs with attention to value enhancement. Some studies examine investment, financing, and benefit distribution. For instance, Kotschi analyzed financing models for organic breeding, emphasizing capital operations for sustainable development [26], while He et al. assessed risks in asset-based operations and proposed preventive measures [27]. These highlight financial support mechanisms but remain macro-level, offering limited insights into the roles of individual links.

Other studies emphasize coordination and organizational models. Nenonen (2019) highlighted that equitable benefit-sharing is critical for stability [28], while Botha (2014) stressed the role of industry platforms in cooperation [29]. Such research underscores governance but is constrained by static analyses that neglect dynamic interactions. Additional work notes the role of intellectual property and market mechanisms. Overall, existing studies provide preliminary insights into OPVR value enhancement but remain fragmented, lacking an integrated framework to explain interactive mechanisms among value chain components.

### 2.4 Value formation mechanisms

Research on value formation mechanisms aims to reveal how value is created, transmitted, and realized, thus linking intellectual property and industrial development. Value formation depends not only on resources and markets but also on institutions and organizational capacity [30]. In agriculture, value chain theory is often used to integrate breeding, production, processing, and marketing into holistic models of value creation and distribution [31]. Building on this, intellectual property has gained attention. Amentae et al. argued that it plays a pivotal role across all agri-food chain stages, from R&D to marketing [32]. Similarly, Greenhalgh (2016) emphasized that IP systems incentivize innovation, lower transaction costs, and improve resource allocation [33]. In seed industry research, few studies examine interactions among value chain components. Gao (2014) reviewed applications of system dynamics (SD) in agricultural value chains, highlighting production–market interactions. Donovan et al. (2021) analyzed from a private-sector view how improved seeds enhance smallholder value.

While these studies illuminate certain links, they remain fragmented, lacking unified frameworks and insufficiently addressing causal relationships or dynamic evolution. Therefore, integrating value chain theory with intellectual property characteristics is essential for studying OPVR. Such integration can clarify internal logic of value creation, transmission, and realization, and provide theoretical support for targeted policies and industry practices.

## 3 Research framework and methods

### 3.1 Research framework

Drawing on the Web of Science database, this study initially identified 15 factors influencing the value of ornamental plant variety rights (OPVR) through a systematic literature review. These factors were further refined via a questionnaire survey, resulting in 12 key determinants. Building on OPVR value chain theory, an evaluation index system was then established. Subsequently, a structural equation model (SEM) was employed to analyze the interrelationships among influencing factors and to uncover the mechanisms of value formation. Finally, a system dynamics (SD) model was developed to capture the dynamic evolution of OPVR value and to assess the effects of different factors, followed by the formulation of value-enhancement strategies.

### 3.2 Structural equation model (SEM)

Structural equation modeling (SEM) is a widely applied method for estimating and testing causal relationships [34]. It integrates observable variables with latent constructs that cannot be directly measured. Unlike traditional analytical approaches, SEM enables simultaneous assessment of multiple relationships among latent variables. By evaluating the significance and relative contributions of each construct, SEM provides a comprehensive understanding of the underlying mechanisms. This approach is particularly suitable for constructing complex, multidimensional conceptual models [35–37]. Given that OPVR value is shaped by the interplay of diverse and interdependent factors, SEM offers a robust framework for examining the interactions between latent and manifest variables in the value formation process.

### 3.3 System dynamics (SD) model

System Dynamics (SD) System dynamics (SD) is a simulation-based methodology that combines qualitative reasoning with quantitative analysis, underpinned by cybernetics, information theory, and decision theory [38]. By constructing mathematical models and simulations, SD investigates the feedback relationships between system behavior and internal mechanisms, often represented through causal loop diagrams (CLDs) and stock–flow diagrams. This method is particularly effective for analyzing the dynamic impacts of multiple factors on OPVR value [39]. In this study, SD modeling revealed that OPVR value is jointly shaped by four dimensions: variety value (Var), technological level (Tec), market and brand (Mar), and intellectual property protection (IPP). The interactions among these factors elucidate the intrinsic mechanisms driving OPVR value formation.

## 4 Process and results

### 4.1 Construction of the OPVR value indicator system

#### 4.1.1 Preliminary identification of factors influencing the OPVR value.

This paper conducts a comprehensive literature review on the determinants influencing the OPVR value, utilizing databases such as Google Scholar and Web of Science. The search employs keywords including “ornamental plant variety rights value”, “influencing factor”, “Value influencing factor”, and “the OPVR value”, adhering to the criteria of high citation frequency and substantial relevance for the analysis of literature. Followingly, we examine the relevant literature, while integrating the current research landscape and the actual circumstances surrounding “the OPVR value formation factors” in the current academic circles. Additionally, we refer to findings related to value influencing factors in agriculture to preliminarily identify the influencing factors of the OPVR value formation and summarize the representative factors identified (see Table 1).

**Table 1:** Identification of influencing factors and treatment results.

Serial number	Influencing factors	The outcome of the process
1	Variety Quality [40,41]	1 Variety quality
2	Carbon Sequestration Capacity [42–44]	removing
3	Consumer Demand [45]	2 Reservations
4	Market Size [46]	3 Reservations
5	Technical Barrier [47,48]	4 Reservations
6	Technical Maturity [47]	5 reservations
7	Technical Stability [49]	removing
8	Production Cost [50]	6 Reservations
9	Logistics System [51]	7 Reservations
10	Flower Language Imagery [52]	8 Combined as Flower language imagery
11	Brand Building [51,53]	
12	Marketing Platform [54]	9 Reservations
13	Protection Policy [50,55]	10 reservations
14	Law Enforcement [50]	11 Reservations
15	Public Awareness [56,57]	12 reservations

#### 4.1.2 Factors influencing the value of OPVR.

This study uses the expert consultation approach to optimize the previously identified factors influencing the OPVR value. First, the definition of the value of ornamental plant varieties was clarified, and the significance and context of this value were explicitly communicated to the experts to assure their comprehension of the objective and scope of the study. The experts were requested to evaluate the previously identified factors to determine which are most significant in influencing the OPVR value. They were also required to complete a questionnaire detailing their perspectives and justifications (refer to S1 Table in S1 File for the questionnaire), and subsequently consolidate the experts’ comments and suggestions. The outcomes of the expert consultation were ultimately assessed statistically based on the frequency of mention. The formula for calculating the degree of mention is A = f/N, where A represents the degree of mention, f denotes the frequency of a factor’s mention, and N signifies the total number of mentions (total number of experts). Upon calculating the mentions of each influential factor, the factors influencing the OPVR value were conclusively established based on the criterion of ≥50% mentions.

The construction of OPVR value is influenced by various sectors; therefore, it is essential to integrate specialists from diverse professional backgrounds during consultations to guarantee the comprehensiveness and reliability of factor selection. Consequently, ten experts were chosen for consultation in this study, comprising research and development personnel, managers from seed industry enterprises, and academic specialists involved in the research of new ornamental plant varieties in universities. The composition of the experts is presented in S2 Table in S1 File.

The outcomes of this consultation were aggregated to obtain mentions of the factors influencing the OPVR value (see S3 Table in S1 File for results). Of the 15 initially identified factors, two-technical stability (Mention = 40%) and Carbon Sequestration Capacity (Mention = 40%) were excluded due to that their mentions are below 50%. The experts’ opinions were summarized as follows: Brand Building and Flower Language Imagery were integrated into Flower language imagery.

Following an extensive review of expert opinions, the factors influencing the OPVR value were integrated and summarized, resulting in the identification of 12 principal factors influencing the OPVR value (refer to the third column of Table 1).

#### 4.1.3 Establishment of the OPVR value evaluation index system.

**Fig 1:**
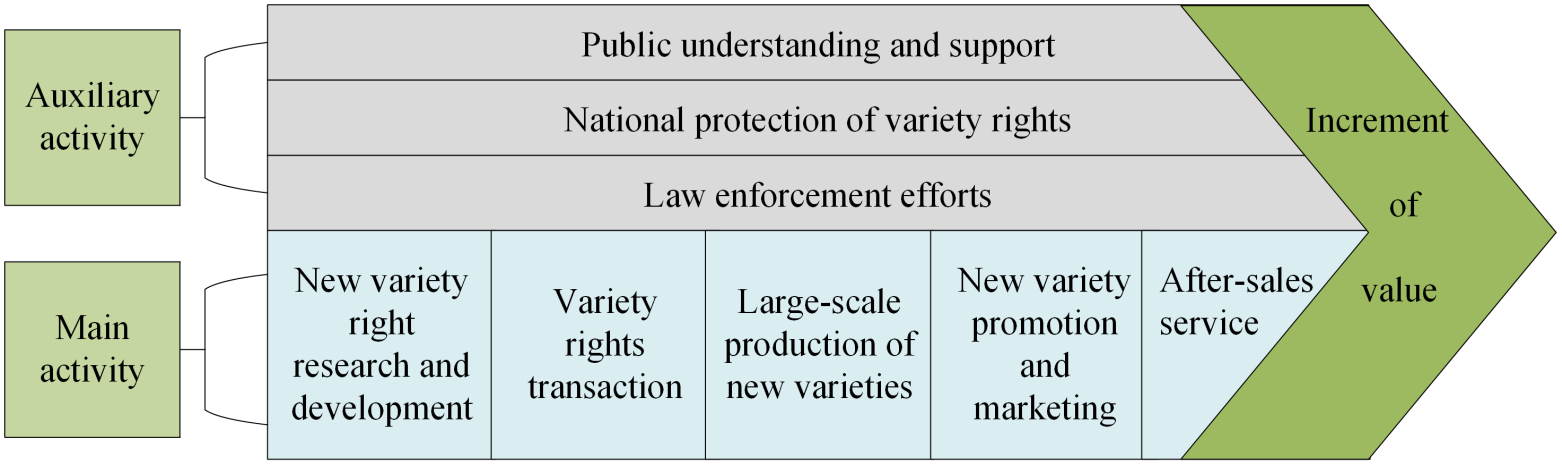
Value chain model of the OPVR.

The value chain analysis model is a prevalent instrument for evaluating and appraising the value created by activities within a company. By examining the cost and value creation capacity of each segment in the value chain, an organization may identify its strengths and weaknesses within each link, thereby uncovering possibilities to enhance value addition and minimize expenses. The value chain of the OPVR encompasses breeding, transfer, propagation, promotion, and sales, with each component being interdependent, designed to optimize the value of plant variety rights and enhance the interests of each functional entity. Based on the preceding analysis, the OPVR value chain model is illustrated in Fig 1.

Through the analysis of the value chain model of the OPVR, this study subdivided the value formation process of the OPVR into three primary stages: development and innovation, expansion and promotion, and commercial utilization.

According to the stage division of the OPVR value chain, this study divides the factors influencing the OPVR value into four categories: Var (Consumer Demand, Variety Quality, Market Size), Tec (Technological Barrier, Technological Maturity, Production Cost), Mar (Flower Language Imagery, Marketing Platform, Logistics System), and IPP (Public Awareness, Protection Policy, Law Enforcement). Ultimately establishing the OPVR value indicator system.

### 4.2 Construction of the SEM

#### 4.2.1 The analysis of factors affecting the OPVR value.

(1) Design, distribution, and analysis of questionnaires

This study employs a questionnaire to investigate the extent of influence of each factor on the OPVR value. To ensure that the questionnaire design is scientific and rational, it must follow the principles of purpose, objectivity and simplicity. The questionnaire encompasses two primary components: the first part gathers fundamental information about the participants, while the second section constitutes the core content, which involves evaluating the significance of each factor’s influence on the OPVR value. The questionnaire utilized a Likert scale, categorizing the significance of influencing factors into five grades: 1 indicates minimal influence, 2 denotes slight influence, 3 represents moderate influence, 4 signifies substantial influence, and 5 reflects considerable influence. The questionnaire is elaborated in S4 Table in S1 File.

A total of 235 questionnaires were collected. To ensure data authenticity and validity, responses with excessively short completion times and invalid questionnaires were excluded, yielding 220 valid samples and an effective response rate of 93.62%. Respondents were primarily from seed enterprises and university research institutions (S5 Table in S1 File). In terms of age, the survey encompassed groups aged 20–30, 31–40, 41–50, and above 50, covering both early-career professionals and senior experts. Regarding education, respondents held bachelor’s to doctoral degrees (S2 Table in S1 File), indicating generally high professional competence. In terms of occupation, the sample included researchers, research managers, variety rights administrators, department heads, and staff (S1 and S2 Tables in S1 File), reflecting the diversity of roles and hierarchies within the industry. This respondent structure enhances the scientific robustness of the findings. Nevertheless, as noted in the “Limitations” section, the sample is largely concentrated on highly educated professionals. Future research should extend to broader groups, including consumers and policymakers, to strengthen the generalizability of the conclusions.

Descriptive statistics are crucial for assessing the appropriateness of sample data and its impact on subsequent reliability and validity evaluations. The computation of kurtosis and skewness indicators facilitates an in-depth evaluation of the distributional patterns of the data. According to research studies, data can be considered to approximately follow a normal distribution when the kurtosis value is below 10 and the skewness value remains below 3. This prerequisite is essential for ensuring an accurate evaluation of reliability and validity, as non-normal data often result in biased or misleading analytical outcomes. The sample data were examined using descriptive statistics in SPSS 27, with the findings presented in S6 Table in S1 File. The table reveals that the absolute values of kurtosis for the questionnaire data are all below 10 and the absolute values of skewness are all below 3. This indicates that the questionnaire data in this study approximately adhere to a normal distribution, making it appropriate for subsequent reliability and validity analyses.

Ethical considerations: This study aims to reveal the mechanism underlying the value formation of Ornamental Plant Variety Rights (OPVR) and to explore optimized pathways for enhancing OPVR value. Prior to participation, all respondents were fully informed of the study’s purpose, procedures, and their rights. Participants provided data based on their professional understanding of the social acceptance, market value, and related indicators of new ornamental plant varieties. Verbal informed consent was obtained before distributing the questionnaire: the researchers explained the study information to each participant, and the questionnaire was issued only after verbal agreement was received. Completion of the questionnaire was further regarded as confirmation of consent. Participation was entirely voluntary, and respondents could withdraw at any time without penalty. As the participants were professionals and the study did not involve vulnerable populations or any sensitive health-related data, no additional written documentation or external witnessing was required. According to national regulations and the standard policies implemented by Chinese universities, such low-risk social science surveys are exempt from formal ethical review, and verbal consent accompanied by appropriate documentation is considered compliant. The sample size met statistical requirements: a total of 235 questionnaires were distributed and collected, with 15 excluded as invalid responses.

(2) Exploratory factor analysis

Exploratory Factor Analysis (EFA) is a statistical technique employed to discern the underlying structure inside a collection of observable variables, primarily aimed at identifying common factors and achieving dimensionality reduction of the data. Prior to conducting exploratory factor analysis on questionnaire data, it is necessary to perform confidence and validation analyses of the data.

This study employed SPSS 27.0 software to perform reliability and validity tests, as well as principal component analysis, on the questionnaire data. The outcomes of the reliability assessment are presented in S7 Table in S1 File. The analysis of the table reveals that the overall Cronbach’s alpha coefficient for the questionnaire data is 0.801, exceeding the threshold of 0.8, while the CITC values for the examined items surpass 0.4, demonstrating high data reliability suitable for further analysis. The outcomes of the validity test are demonstrated in S8 and S9 Tables in S1 File. The analysis of the table reveals that the KMO value for each potential variable in both the overall and sample data of the questionnaire exceeds 0.7, while the P-value is 0.000, which is below 0.05, is suitable for information extraction, and the validity results are superior, aligning with the prerequisite criteria for conducting factor analysis. The questionnaire data underwent principal component analysis using SPSS27.0, after reliability and validity testing to satisfy the requirements. S10 Table in S1 File demonstrates the factor rotated component matrix of the questionnaire data from this study. The cumulative variance explained rate is 83.014%, exceeding 50%, indicating a high level of structural validity. All assessed items exhibited considerable loading on the corresponding variables, with a commonality over 0.4, confirming the validity of the information extraction.

(3) Validation factor analysis

Confirmatory Factor Analysis (CFA) is typically employed to assess the extent to which a theoretical model aligns with the observed data, specifically evaluating the fit between the SEM and the data.

In conjunction with the prior analysis, four potential variables in the SEM of the OPVR value are identified, namely, Var, Tec, Mar, and IPP. Each latent variable is associated with three observation variables, twelve error variables, and four disturbance variables. The detailed descriptions of the model variables are included in Table 2 below.

**Table 2:** Clarification of SEM variables.

Latent Variable	Observational Variable	Disturbing Variance	Confounder
Var	Consumer Demand (Var_1_)	e1	e13
	Variety Quality (Var_2_)	e2	
	Market Size (Var_3_)	e3	
Tec	Technical Barrier (Tec_1_)	e4	e14
	Technical Maturity (Tec_2_)	e5	
	Production Cost (Tec_3_)	e6	
Mar	Flower Language Imagery (Mar_1_)	e7	e15
	Marketing Platform (Mar_2_)	e8	
	Logistics System (Mar_3_)	e9	
IPP	Public Awareness (IPP_1_)	e10	e16
	Protection Policy (IPP_2_)	e11	
	Law Enforcement (IPP_3_)	e12	

**Fig 2:**
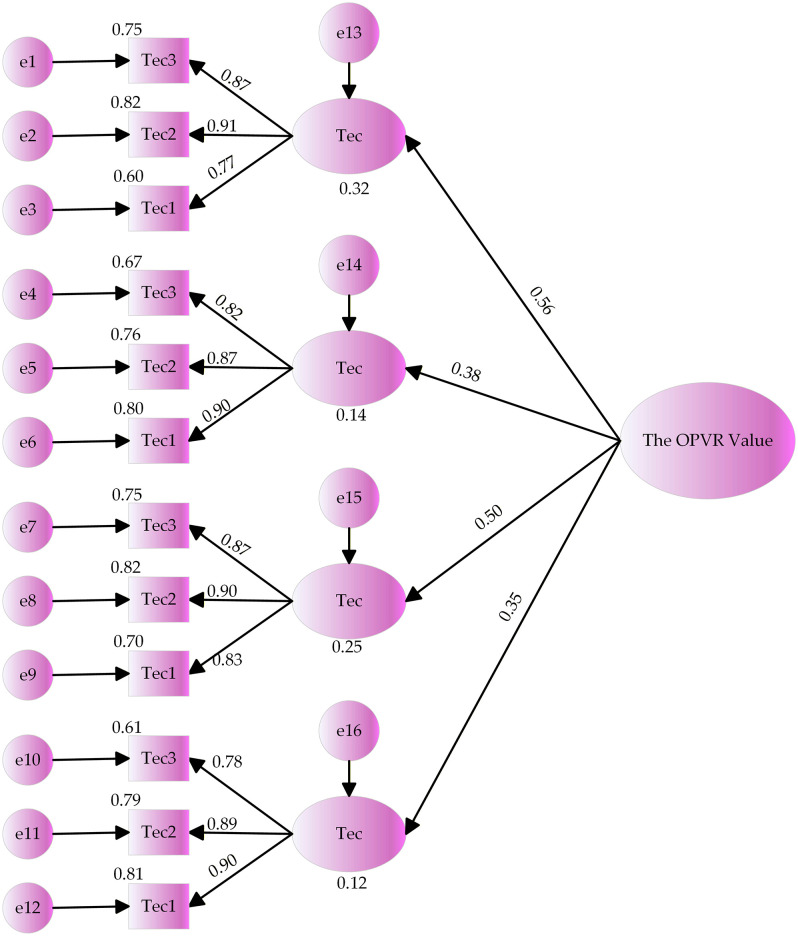
Model fitting results of influencing factors on the OPVR value.

The collected questionnaire data was imported into each variable in AMOS 29.0 and the run results are depicted in Fig 2.

The model fit metric is a critical metric in SEM to assess how well the model fits the data (S11 Table in S1 File). The analysis of the table reveals that all of the fit indicators are within the acceptable range, the model is well-fitted. Fig 2 illustrates the expression of the OPVR value as:


 the OPVR value=0.56*Var+0.38*Tec+0.50*Mar+0.35*IPP (1)

The expression for normalizing the coefficients in the aforementioned equation is as follows:


 the OPVR value=0.31*Var+0.20*Tec+0.30*Mar+0.19*IPP (2)

#### 4.2.2 Analysis of the interaction between influencing factors.

(1) Assumptions on the relationship between the roles of the influencing factors

The following hypothesis is proposed regarding the interaction relationship between the factors that influence the OPVR value, based on the SEM constructed above, the relevant literature, and the actual situation:

H1: Var has a significant positive effect on Tec.

H2: Var has a significant positive effect on Mar.

H3: Tec has a significant positive effect on Mar.

H4: IPP has a significant positive effect on Var.

H5: IPP has a significant positive effect on Tec.

H6: IPP has a significant positive effect on Mar.

**Fig 3:**
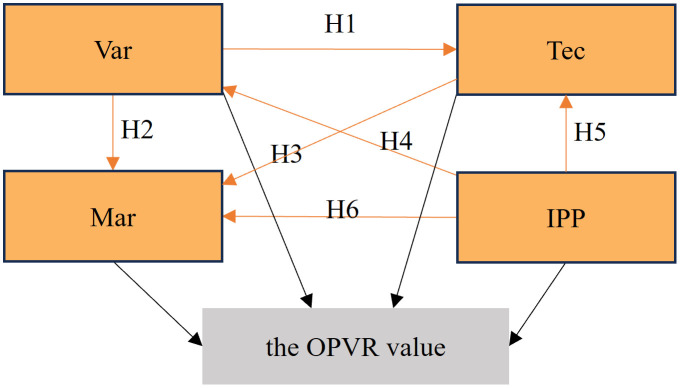
Hypothetical model for the study of the OPVR value.

Therefore, the hypothetical model for the study of the OPVR value is as follows (Fig 3).

(2) Initial modeling and testing

**Fig 4:**
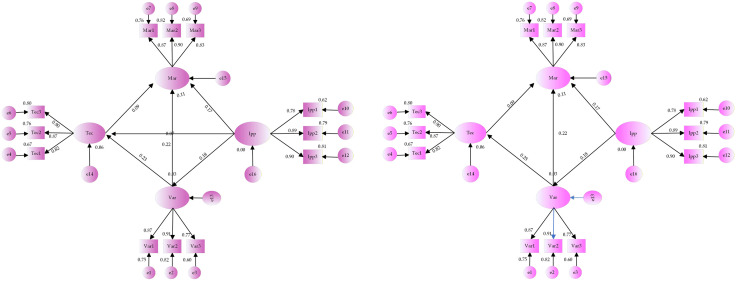
(a) Preliminary hypothetical model fitting results of the relationship between influencing factors of the OPVR value. (b) primary revised structural model diagram of the relationship between influencing factors of the OPVR value.

The preliminary hypothetical model delineating the relational dynamics among the factors influencing the OPVR value is constructed, as illustrated in Fig 4a, combined with the proposed hypothetical relationship among the factors influencing the OPVR value depicted in Fig 3.

Model fitting calculations were performed in AMOS29.0 software. The initial hypothesized model overall fit calculations were conducted first (S12 Table in S1 File). The fit metrics were all within acceptable ranges, and the model fit was satisfactory. Then, the intrinsic structural fit of the initial hypothetical model was calculated (refer to S13 Table in S1 File). The results demonstrated that the combined reliability (CR) of each potential variable was greater than 0.7, and the average variance extracted (average variance extracted AVE) was greater than 0.5. These findings suggested that the intrinsic structural fit of the initial model was satisfactory, and it possessed a high degree of stability and reliability. Fig 4a illustrates the preliminary findings.

(3) Modification of the model

This study evaluates the proposed hypothesis by assessing the significance of the postulated routes, specifically determining if the p-value is below 0.05. Table 3 presents. the standardized coefficients of the paths from the initial hypothesized model and the results of the correlation test. The p-values for the paths “Mar← Tec” and “ Tec← IPP” exceed 0.05, indicating that the hypotheses for these two paths are unsupported and the initial model needs to be corrected as necessary.

**Table 3:** Path coefficient of the initial hypothesis model and results of significance testing.

Hypothesis number	Hypothetical path	Standardized path coefficient	C.R.	P-value	Path supported or not
H1	Tec ← Var	0.230	3.052	0.002	Yes
H2	Mar ← Var	0.220	2.906	0.004	Yes
H3	Mar ← Tec	0.090	1.254	0.210	No
H4	Var ← IPP	0.180	2.351	0.019	Yes
H5	Tec ← IPP	0.067	0.873	0.383	No
H6	Mar ← IPP	0.170	2.317	0.021	Yes

**Fig 5:**
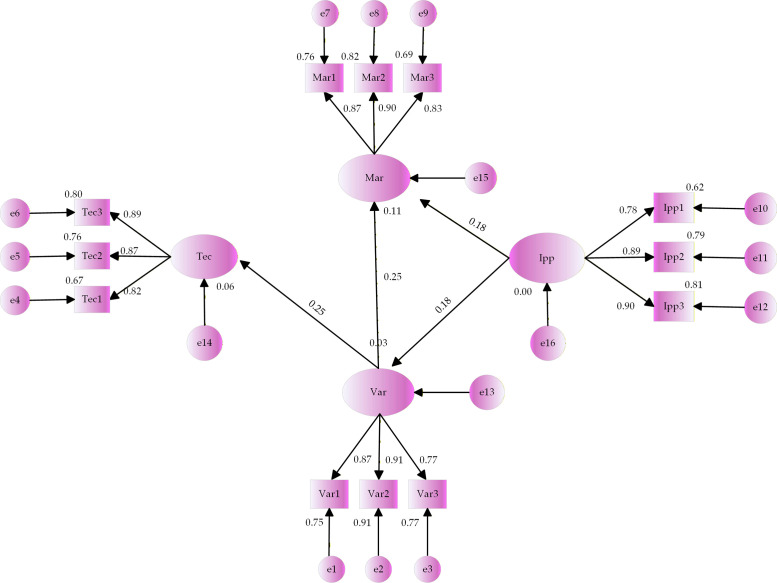
The second revised structural model diagram of the relationship between factors influencing the OPVR value.

According to Table 3, the p-value for the two paths “Mar← Tec” and “Tec← IPP” exceeds 0.05, failing to meet the significance criterion; thus, hypotheses H3 and H5 cannot be supported. Therefore, in this study, adhering to the SEM principle, the paths were sequentially corrected, beginning with the elimination of the least significant hypothesis path “Tec← IPP” to obtain the modified structural model depicting the components impacting the factors influencing the OPVR value (Fig 4b). The modified model underwent a goodness-of-fit test (S14 Table in S1 File), with all fit indicators falling within the acceptable range, indicating a satisfactory fit. The calculation of path coefficients and their significance was conducted (S15 Table in S1 File), revealing that the removal of “Tec← IPP” from the original model did not render the path “Mar← Tec” significant. This suggests that hypothesis H3 remains invalid. Therefore, in this study, the model underwent two modifications by removing the path “Tec← IPP”, resulting in a second modified structural model illustrating the relationship between the factors affecting the OPVR value (Fig 5). The assessment of the fit of the model indicates that the overall fit and internal structure of the second revised model are superior. Therefore, the second revised structural model delineating the factors influencing the OPVR value becomes the definitive model of this study.

#### 4.2.3 Analysis of the mechanism underlying the formation of the OPVR value.

**Fig 6:**
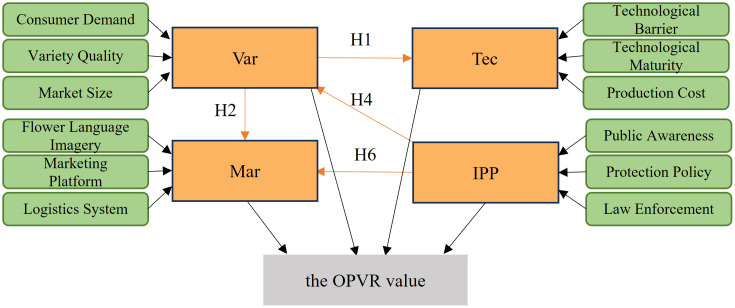
Model diagram of the formation mechanism of the OPVR value.

The questionnaire was designed, distributed, and retrieved, utilizing the previously established factors affecting the OPVR value to conduct a factor analysis of the influencing factors. the SEM is constructed using AMOS 29.0 by integrating questionnaire data, theoretical research, and empirical conditions. The hypothesis model is established, followed by a path significance test, and the model is corrected based on the test results. Finalize the model diagram of the formation mechanism of the OPVR value (Fig 6).

The findings indicate that the contributions of four types of influencing factors to the formation of the OPVR value are as follows: Var is 0.31, Tec is 0.20, Mar is 0.30, and IPP is 0.19. The results also show that Var is affected by IPP, “consumer demand”, “variety quality”, and “market scale”; Tec is affected by Var, “technology barriers”, ”technology maturity”, and “production costs”; Mar is affected by Var and IPP, as well as “floral imagery”, “marketing platforms”, and “logistics systems”. The mechanism model of the OPVR value established herein and the computed standardized path coefficient provide the basis for the subsequent establishment of the SD model.

### 4.3 Investigation of strategies for enhancing the OPVR value based on SD model

#### 4.3.1 Analysis of SD model of the OPVR value.

**Fig 7:**
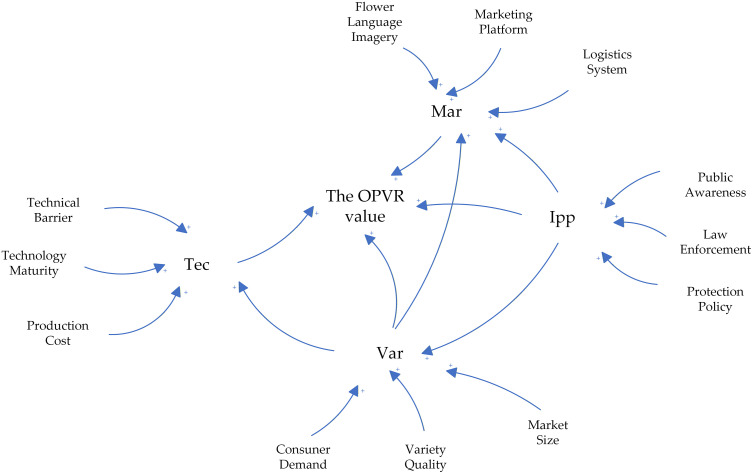
Causal loop diagram illustrating the OPVR value.

Based on SD theory, a SD causality diagram of the OPVR value is constructed, as shown in Fig 7.

An in-depth examination of the causality diagram for the OPVR value yielded the subsequent feedback routes:

(1) Consumer Demand, Variety Quality, etc. → + Var → + the OPVR value
(2) Technical Barrier, Technical Maturity, etc. → + Tec → + the OPVR value
(3) Flower Language Imagery, Marketing Platform, etc. → + Mar → + the OPVR value
(4) Public awareness, Law enforcement, etc. → + IPP → + the OPVR value
(5) Var → + Tec → + the OPVR value
(6) Var → + Mar → + the OPVR value
(7) IPP → + Var → + the OPVR value
(8) IPP → + Var → + Tec → + the OPVR value
(9) IPP → + Var → +Mar → + the OPVR value
(10) IPP → + Mar → + the OPVR value

In conjunction with the previous analysis of the factors affecting the OPVR value and existing research, the diverse variable types in the simulation model of the OPVR value were determined, presented in S16-S18 Tables in S1 File.

**Fig 8:**
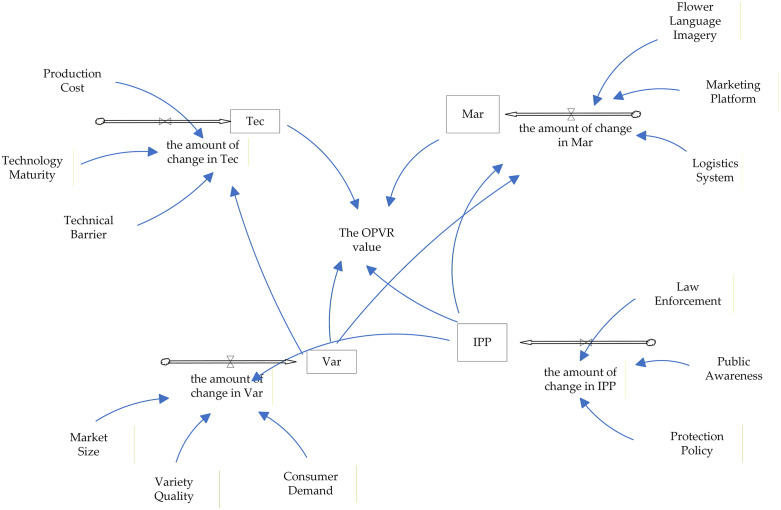
Stock flow diagram of the OPVR value system.

The integration of the aforementioned variables and causality diagrams to construct a stock-flow diagram of the OPVR value system (Fig 8).

In conjunction with the SEM of the OPVR value constructed in the article, the standard path coefficients and factor loadings of the potential and observed variables are normalized to serve as the weights of the variables in the SD model, with the calculation algorithm delineated as follows:


$$W_{i}=\frac{\gamma_{i}}{\sum_{i=\text{1}}^{n}\gamma_{i}}$$ (3)

where W_i_ represents the normalized weight coefficient of the variable and γ_i_ denotes the standard path coefficient of the indicated or observed variable. The results of the calculations are shown in Table 4.

**Table 4:** Standardized path coefficients of factors influencing the OPVR value.

Level 1 indicators	Standardized path factor	Normalized weight	Secondary indicators	Standardized path factor	Normalized weight
Var	0.56	0.31	Consumer Demand	0.867	0.34
			Variety Quality	0.907	0.356
			Market Size	0.775	0.304
Tec	0.38	0.212	Technical Barrier	0.82	0.317
			Technical Maturity	0.871	0.337
			Production Cost	0.896	0.346
Mar	0.5	0.279	Flower Language Imagery	0.868	0.333
			Marketing Platform	0.903	0.347
			Logistics System	0.834	0.32
IPP	0.35	0.196	Public Awareness	0.784	0.305
			Protection Policy	0.889	0.346
			Law Enforcement	0.899	0.349

In addition, as illustrated above, there exist indirect effects among the potential variables, with the corresponding weights derived from the calculation of these indirect effects presented. the normalized weights are as follows: Var → Tec is 0.091, Var → Mar is 0.074, IPP → Var is 0.065, IPP → Mar is 0.052.

In SD, the initial state-level value of a variable represents the value assumed by the system at the outset. The initial value of the state variable may be a forecasted value, a historical value or another type. This paper utilizes the weighted average of the expert scoring data, in conjunction with relevant literature, as the initial value of the state variable. The variable values are as follows: Var factor status value is 4.415, Tec factor status value is 3.272, Mar factor status value is 2.963, IPP factor status value is 3.423.

According to the SEM of the formation mechanism of the OPVR value established in the article, and considering the actual situation and pertinent research, the initial time of the model is established at 0, the simulation duration is set to 10 years, and the simulation step size is defined as 1 month. The formulated SD equations are as follows:

FINAL TIME = 120, Units: Month; INITIAL TIME = 0, Units: Month;

TIME STEP = 1, Units: Month

Amount of change of Var = 0.065*IPP + 0.935*(0.34*Consumer Demand + 0.356*Variety Quality + 0.304*Market Size)

Amount of change of Tec = 0.091*Var + 0.909*(0.317*Technological Barrier + 0.337*Technological Maturity + 0.346*Production Cost)

Amount of change of Mar = 0.074* Var + 0.052* IPP + 0.874* (0.333* Flower Language Imagery + 0.347* Marketing Platform + 0.32* Logistics System)

Amount of change of IPP = 0.305*Public Awareness + 0.346*Protection Policy + 0.349* Law Enforcement

The OPVR Value = 0.313*Var + 0.212*Tec + 0.279*Mar + 0.196*IPP

#### 4.3.2 Examination of the mechanism underlying the formation of the OPVR value.

(1) Model runs and analysis

The previously identified SD equations were incorporated into the model, and the SEM factor loadings were as initial values for the variables in the SD model for trend prediction, the simulation duration was set to 10 years, with the prediction results illustrated in S1 Fig in S1 File.

The OPVR value has demonstrated consistent and exponential growth throughout the 10-year simulation period. Specifically, in the first four years of the simulation period, the OPVR value remained low and the growth rate was minimal. This was primarily attributed to the instability of production technology for new ornamental plant varieties and the lack of significant marketing outcomes in the preceding period, leading to limited market awareness and public knowledge, which in turn resulted in a negligible enhancement of the OPVR value. However, in the six years subsequent to the fourth year, the expansion of marketing coverage, ongoing stabilization and enhancement of production technology, and the improvements in public awareness have collectively contributed to a significant increase in, the OPVR value.

(2) Sensitivity test of factors affecting the OPVR value

1) The initial values of the four primary modules influencing the OPVR value were modified, and comparative simulations were conducted accordingly.

Program 1: 30% increase in the input value of Var; Program 2: 30% increase in the input value of Tec.

Program 3: 30% increase in the input value of Mar; Program 4: 30% increase in the input value of IPP.

**Fig 9:**
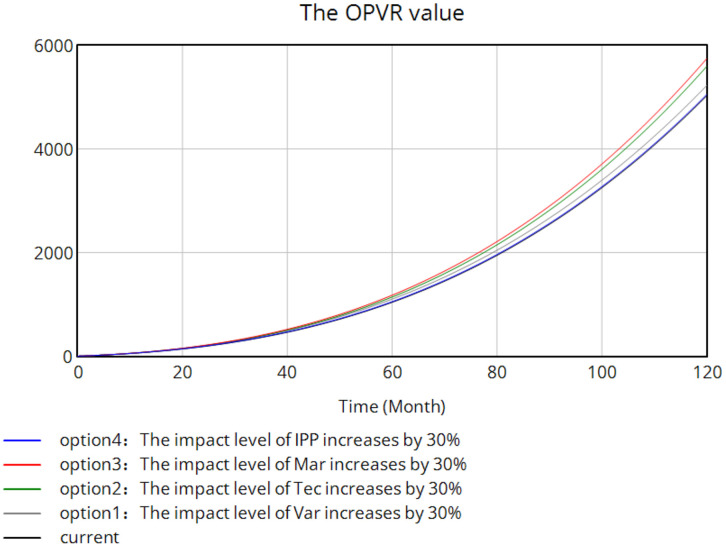
Prediction of the trend of the impact of primary indicators on the OPVR value.

The aforementioned scenarios are sequentially input into the SD model for simulation, with the resulting trend graph presented in Fig 9. The figure illustrates that the adjustment of various influencing factors affects the OPVR value to different extents. During the initial four years of the simulation period, the variations in the influence of various factors on the OPVR value are not significant. However, after this period, distinctions begin to emerge, with the impact on the OPVR value ranked as follows: Mar > Tec > Var > IPP. Among them, a 30% increase in the input value of Mar significantly impacts the enhancement of the OPVR value, with effects becoming more pronounced over time. This suggests that the effect of Mar on the OPVR value enhancement requires a longer duration for accumulation. Additionally, the brand awareness can be enhanced through effective branding and appropriate marketing strategies, which can elevate the added value of the ornamental plant varieties, foster consumer loyalty and increase the overall value of ornamental plants. Brand building and the adoption of appropriate marketing strategies can enhance the brand awareness, increase the added value of new ornamental plant varieties, and establish consumer loyalty. This, in turn, promotes the sales of these new varieties and improves their market competitiveness and value.

2) The study adjust the initial values of the four major modules of influencing factors and conduct comparative simulations.

The influence of alterations on the OPVR value was simulated by modifying individual factors within the four modules of Var, Tec, Mar, and IPP respectively, by an increase of 30% from the original value. Various scenarios were sequentially entered into the SD model of the OPVR value for simulation, with the results presented in S19-S22 Tables in S1 File. They yielded the following conclusions:

The three Var factors—Variety Quality, Market Size, Consumer Demand—all significantly boost the OPVR value, with their influence ranked as follows: Variety Quality > Consumer Demand > Market Size.

The three factors of Technical Barriers, Technological Maturity, and Production Cost included in the Tec factor exert a certain effect on the OPVR value, with negligible significance and indistinct differences among the three components. The hierarchy of effects is as follows: Technical Barrier > Production Cost > Technological Maturity.

The three factors of Mar—Flower Language Imagery, Marketing Platform, and Logistics System—significantly influence the enhancement of the OPVR value, with the Marketing Platform exerting the most pronounced effect.

Public Awareness, Protection Policy, and Law Enforcement of IPP profoundly influence the OPVR value, especially the Protection Policy and Law Enforcement aspects.

### 4.4 Results

This study explores the strategies to enhance the OPVR value from four aspects: Var, Tec, Mar, and IPP, in conjunction with the findings of SD simulation.

(1) In terms of Var: The enhancement of quality in new varieties is the core of breeding; it is essential to enhance ornamental value and adaptability, employ advanced technology to cultivate plant varieties with vibrant colors, and extended flowering periods, benefit the ecological environment, and establish a rigorous quality control system. Additionally, it is recommended to concentrate on consumer demand, conduct market research prior to research and development, and identify new varieties’ the target market and positioning, to improve market competitiveness and consumer satisfaction, and further maximize OPVR value. Furthermore, we define the market size, analyze the competitive landscape, predict market development trend, and comprehend demand through horticultural and plant exhibitions and other channels, thereby facilitating varietal improvement and strategic marketing.
(2) In terms of Tec: The simulation results of the technology level module indicate that its effect on the OPVR value is ranked as follows: Technical barriers, Production cost, and Technological maturity. Establishing technical barriers is necessary for seed industry enterprises to improve their core competitiveness. They should also strengthen market research, introduce high-quality germplasm resources train top talent, and strengthen technical monitoring and legal protection of new varieties in order to create technical advantages. In order to improve the resilience and yield of new varieties and decrease losses from diseases and adversities, we need to develop sensible production plans and cost control strategies, enhance automation, and implement cutting-edge forestry technology and innovative techniques. To enhance the technical maturity and marketability of the ornamental plant varieties, interdisciplinary collaboration and field verification should also be strengthened.
(3) In terms of Mar: For the OPVR value, the strategy in terms of Mar emphasizes the establishment of logistics systems, the creation of floral symbolism and the development of marketing platforms. Collaborating with breeders to establish a professional information website and improve the information promotion path is recommended. In addition, an online service platform should be built to expand sales channels and enhance brand promotion. The construction of the logistics system should be strengthened to ensure strict, efficient and punctual characteristics, manage and optimize the supply chain, and build a seamless flower circulation system. To ensure consistent quality of flower varieties, it is imperative to enhance the cold chain management to sustain optimal temperature and humidity levels. To develop flower language imagery, the ornamental plants for this special category, the seed industry should incorporate the characteristics of flowers and their emotional symbolism within cultural contexts to establish a profound emotional connection with the consumers, thereby augmenting the value-added and brand competitiveness of the flowers.
(4) In terms of IPP: The simulation results of the IPP module indicate that the Protection policy, Law enforcement and Public awareness all place a positive effect on the OPVR value. These impacts are in reverse order. In this regard, the associated strategies are proposed: to increase the protection of the OPVR, the pertinent laws and regulations must be enforced and the level of intellectual property rights protection of plant varieties must be raised in light of global experience. To encourage innovation and equitable competition among plant breeders, it is essential to strengthen law enforcement, refine the organization of law enforcement departments, clarify the responsibilities and authorities of law enforcement, establish a robust law enforcement mechanism, elevate the standardization and transparency of law enforcement, impose stringent penalties for infringement, and effectively safeguard the legitimate rights and interests of breeders. We should also improve the dissemination of intellectual property rights, and raise public understanding regarding the need of protecting plant varieties.

## 5 Conclusions and discusses

### 5.1 Main findings and discussion

This study identified four categories of factors influencing OPVR value—variety value (Var), technological level (Tec), market and branding (Mar), and intellectual property protection (IPP)—and applied SEM and SD models to reveal their causal mechanisms and dynamic evolution.

First, SEM results showed that the contributions ranked as Var (0.31)> Mar (0.30)> Tec (0.20)> IPP (0.19). Var not only directly enhanced value but also indirectly promoted Tec and Mar. Although IPP contributed least, it positively influenced both Var and Mar. Path analysis indicated that key drivers of Var were variety quality, consumer demand, and market scale; Tec was limited by technological barriers, production costs, and maturity; Mar was shaped by marketing platforms, symbolic attributes of flowers, and logistics; IPP was driven by policy protection, enforcement, and public awareness.

The SD model, based on causal loop and stock–flow diagrams, highlighted OPVR dynamics. During the first four simulation years, factor impacts were similar, but differences emerged thereafter, with sensitivity ranking: Mar > Tec > Var > IPP. Within each category, sensitivity orders were: Var (variety quality > consumer demand > market scale), Tec (technological barriers > production costs > maturity), Mar (marketing platforms most sensitive), and IPP (policy protection and enforcement > public awareness).

Overall, SEM emphasized static causal relationships, whereas SD revealed dynamic shifts. Market effects were weak early but became decisive over time; technological effects accumulated steadily; variety and policy factors formed the foundation, with differentiated roles across value chain stages.

From these results, three main insights can be drawn:

(1) Variety and demand factors: SEM confirmed that Var had the strongest direct and indirect effects, with variety quality and consumer demand as core drivers. This aligns with U.S. studies [57], where household spending drove market expansion. In China, however, new varieties often fail to achieve premium value unless aligned with consumer demand [58], underscoring the need to couple supply-side innovation with preferences in developing markets.
(2) Market and consumption factors: The SD model showed that Mar became most sensitive after four years, reflecting delayed consumer acceptance and recognition. This is consistent with [59], which stressed consumer trends and marketing strategies as long-term value drivers. By contrast, China faces weak commercialization, slow brand building, and limited awareness [58]. Thus, the late emergence of market effects reflects lagging consumer awareness and underdeveloped branding.
(3) Policy and institutional factors: Policy and IPP contributed least, contrasting with Di Vita et al. (2023), who found policy governance critical in Italy’s aromatic plant sector. In China, weak enforcement and low awareness limit policy effects, preventing full realization of policy dividends. This suggests institutional divergence: developing countries often show weak implementation, while developed economies achieve closer coordination between institutions and markets [18].

### 5.2 Market and strategic implications

This study confirms the critical role of market and variety value highlighted in prior research, while further uncovering the lagged effect of market influence and the limited effectiveness of policy enforcement in developing countries. These findings generate several strategic and policy implications for key stakeholders in the ornamental plant industry:

(1) Enterprise level: optimizing market strategies and variety development

SEM and SD results demonstrated that variety value and market demand are the core drivers of OPVR value. Enterprises should strengthen market orientation in R&D by focusing not only on variety quality and differentiation but also on consumer aesthetics and emotional value. Marketing strategies must be continuously adjusted and innovated to align with evolving consumer preferences and behaviors [60].

(2) Government level: enhancing policy support and enforcement

Although IPP contributed relatively little, it exerted positive spillover effects on variety and market factors. Governments should therefore improve enforcement and supervision of variety rights, establish robust certification and traceability systems, and raise public awareness through subsidies, tax incentives, and promotional campaigns. Such measures would safeguard innovation and facilitate commercialization [61], thereby improving the practical effectiveness of policies within the value chain.

(3) Industry organizations and associations: fostering value chain collaboration

Industry associations should play a pivotal role in standardization, information sharing, and international promotion, thereby reducing uncertainties in R&D and marketing. Precision agriculture and ICT applications can be leveraged to establish unified data protocols, standardized terminology, and traceable management systems. Creating an information platform for new ornamental plant varieties would enable more efficient coordination among research institutions, enterprises, and market actors [62]. From a network governance perspective, associations can further promote organizational innovation and cross-actor collaboration, creating mechanisms for joint credit and risk sharing that enhance transparency and predictability [63]. Moreover, drawing on governance experiences from developed countries such as the United States and Italy could strengthen industry-wide structures, promote sustainable development, and enhance international competitiveness in the ornamental plant sector.

### 5.3 Limitations and future directions

This study developed a value chain framework, grounded in existing literature and expert input, to systematically examine the key factors shaping OPVR value. However, research on new variety valuation remains nascent, and case data availability is limited. The reliance on expert interviews and surveys, while useful, cannot entirely eliminate subjectivity. Furthermore, the future introduction of similar ornamental products may erode the competitiveness of existing new varieties, shortening their market life cycles.

Future research may extend along three directions:

(1) Variety iteration factor: Incorporating variety iteration into OPVR value assessment to account for shortened life cycles and better capture the effects of market dynamics.
(2) Cross-country comparisons: Conducting comparative studies between developed and developing countries to test model applicability under different institutional and market contexts and to identify divergences in value formation mechanisms.
(3) Consumer-level data: Integrating large-scale consumer survey data to complement expert interviews and more accurately assess the influence of demand-side preferences on OPVR value.

## Supporting information


S1 File.
**S1 Table.** Questionnaire on Factors Affecting the Formation of the Value of the Ornamental Plant Variety Rights. **S2 Table.** Expert structure. **S3 Table.** Mention degree of influencing factors. **S4 Table.** Questionnaire on the extent to which factors influence the OPVR value. **S5 Table.** Fundamental Statistical Information of Survey Questionnaire. **S6 Table.** Descriptive Statistics of Sample Data Variables (N = 220). **S7 Table.** Reliability test of the survey questionnaire. **S8 Table.** KMO and Bartlett’s test for overall questionnaire data. **S9 Table.** KMO and Bartlett’s test for each variable in the questionnaire data. **S10 Table.** Factor Rotation Component Matrix. **S11 Table.** Adaptability Test for the Second Order Confirmatory Factor Analysis of the OPVR value. **S12 Table.** Initial hypothesis model suitability test. **S13 Table.** Fitting of the Intrinsic Structure of the Initial Hypothesis Model. **S14 Table.** Hypothesis model fitness test following the first revision. **S15 Table.** Path coefficients of the hypothesis model and the results of their significance test results following one modification. **S16 Table.** Details of System State Variables. **S17 Table.** Details of Rate Variables in the Value System of the OPVR. **S18 Table.** List of Auxiliary Variables in the Value System of the OPVR. **S19 Table.** Simulation comparison of variety kernel modules. **S20 Table.** Simulation comparison of variety kernel modules. **S21 Table.** Simulation comparison of marketing module. **S22 Table.** Simulation comparison of intellectual property protection sales module. **S1 Fig.** Prediction of the OPVR value.
(ZIP)
