# Supplementary material for: The mechanism of the ornamental plant variety rights value formation and enhancement strategy based on SEM-SD
Source: PLoS One. 2025 Dec 19;20(12):e0336934. doi: 10.1371/journal.pone.0336934 (PMC12716751; doi:10.1371/journal.pone.0336934)
Supplement: S1 File. — S1 Table. Questionnaire on Factors Affecting the Formation of the Value of the Ornamental Plant Variety Rights. S2 Table. Expert structure. S3 Table. Mention degree of influencing factors. S4 Table. Questionnaire on the extent to which factors influence the OPVR value. S5 Table. Fundamental Statistical Information of Survey Questionnaire. S6 Table. Descriptive Statistics of Sample Data Variables (N = 220). S7 Table. Reliability test of the survey questionnaire. S8 Table. KMO and Bartlett’s test for overall questionnaire data. S9 Table. KMO and Bartlett’s test for each variable in the questionnaire data. S10 Table. Factor Rotation Component Matrix. S11 Table. Adaptability Test for the Second Order Confirmatory Factor Analysis of the OPVR value. S12 Table. Initial hypothesis model suitability test. S13 Table. Fitting of the Intrinsic Structure of the Initial Hypothesis Model. S14 Table. Hypothesis model fitness test following the first revision. S15 Table. Path coefficients of the hypothesis model and the results of their significance test results following one modification. S16 Table. Details of System State Variables. S17 Table. Details of Rate Variables in the Value System of the OPVR. S18 Table. List of Auxiliary Variables in the Value System of the OPVR. S19 Table. Simulation comparison of variety kernel modules. S20 Table. Simulation comparison of variety kernel modules. S21 Table. Simulation comparison of marketing module. S22 Table. Simulation comparison of intellectual property protection sales module. S1 Fig. Prediction of the OPVR value. (ZIP) [file pone.0336934.s001.zip › supporting information/S1 Table.docx]

Questionnaire seeking Expert Insights on Factors Affecting the Formation of the Value of the Ornamental Plant Variety Rights

Esteemed experts:

Greetings! I truly appreciate your willingness to engage in this inquiry despite your demanding schedule. I am a postgraduate student from the Civil Engineering Department of the School of Civil Engineering at Nanjing Forestry University. This questionnaire aims to comprehensively and methodically identify the factors influencing the formation of the value of the Ornamental Plant Variety Rights, as well as to analyze the mechanism by which the value of these rights is established. Given your profound knowledge and extensive practical

| Factor | Selection, modification and addition  (Tick to indicate choice) | Reasons for non-selection and additional comments |
| --- | --- | --- |
| Variety Quality |  |  |
| Consumer Demand |  |  |
| Market Size |  |  |
| Technical Barrier |  |  |
| Carbon Sequestration Capacity |  |  |
| Technical Maturity |  |  |
| Technical Stability |  |  |
| Production Cost |  |  |
| Logistics System |  |  |
| Flower Language Imagery |  |  |
| Brand Building |  |  |
| Marketing Platform |  |  |
| Protection Policy |  |  |
| Law Enforcement |  |  |
| Public Awareness |  |  |

Experience in the field of the Ornamental Plant Variety Rights, we hereby invite you to contribute to our survey and offer your valuable insights. We solemnly affirm that the results of this questionnaire will be exclusively utilized for academic research, and will not be disclosed to the public under any circumstances. I am extremely grateful for your support and involvement! We wish you good luck in your work and happy life!
