# Supplementary material for: The mechanism of the ornamental plant variety rights value formation and enhancement strategy based on SEM-SD
Source: PLoS One. 2025 Dec 19;20(12):e0336934. doi: 10.1371/journal.pone.0336934 (PMC12716751; doi:10.1371/journal.pone.0336934)
Supplement: S1 File. — S1 Table. Questionnaire on Factors Affecting the Formation of the Value of the Ornamental Plant Variety Rights. S2 Table. Expert structure. S3 Table. Mention degree of influencing factors. S4 Table. Questionnaire on the extent to which factors influence the OPVR value. S5 Table. Fundamental Statistical Information of Survey Questionnaire. S6 Table. Descriptive Statistics of Sample Data Variables (N = 220). S7 Table. Reliability test of the survey questionnaire. S8 Table. KMO and Bartlett’s test for overall questionnaire data. S9 Table. KMO and Bartlett’s test for each variable in the questionnaire data. S10 Table. Factor Rotation Component Matrix. S11 Table. Adaptability Test for the Second Order Confirmatory Factor Analysis of the OPVR value. S12 Table. Initial hypothesis model suitability test. S13 Table. Fitting of the Intrinsic Structure of the Initial Hypothesis Model. S14 Table. Hypothesis model fitness test following the first revision. S15 Table. Path coefficients of the hypothesis model and the results of their significance test results following one modification. S16 Table. Details of System State Variables. S17 Table. Details of Rate Variables in the Value System of the OPVR. S18 Table. List of Auxiliary Variables in the Value System of the OPVR. S19 Table. Simulation comparison of variety kernel modules. S20 Table. Simulation comparison of variety kernel modules. S21 Table. Simulation comparison of marketing module. S22 Table. Simulation comparison of intellectual property protection sales module. S1 Fig. Prediction of the OPVR value. (ZIP) [file pone.0336934.s001.zip › supporting information/S4 Table.docx]

Questionnaire regarding the extent to which the value of the Ornamental Plant Variety Rights is influenced by various factors

Dear Respondent:

Greetings! I am a graduate student majoring in civil engineering at the School of Civil Engineering, Nanjing Forestry University. Currently, I am conducting research on the mechanism of formation and the strategy for enhancing the Ornamental Plant Variety Rights(OPVR). The primary objective of this questionnaire survey is to conduct a comprehensive examination of the various factors that affect the OPVR value and the extent of their impact. Through this study, we expect to reveal the mechanism by which the OPVR value is established and to identify effective strategies to enhance this value. This questionnaire consists of two main parts. The first sections emphasize the acquisition and investigation of fundamental information. The second section focuses on the factors influencing the formation of the OPVR value. Kindly compete each section in accordance with the requirements. Our research will be significantly influenced by your insightful comments and recommendations. We guarantee that your responses will be treated with the utmost confide and utilized exclusively for academic research. Thank you very much for your participation and support! Part I: introduction to Basic Information

What is your age?

☐20 to 30 years ☐31 to 40 years ☐41 to 50 years ☐50 years or older

2. What is the highest level of education you have received?

☐ Specialized ☐ Bachelor's ☐ Master's ☐ Doctoral

3. What is your current position?

☐ Researchers ☐ Research managers ☐ Variety rights managers ☐ Heads of units ☐ General staff

4. What is your professional experience?

☐ 0 to 5 years ☐ 6 to 10 years ☐ 11 to 15 years ☐ 15+ years

Section II: Survey of Factors Affecting the Formation of the Value of the Ornamental Plant Variety Rights(OPVR)

This questionnaire is designed using the classic Likert five-level scale to ensure the accuracy and effectiveness of the assessment. It lists 12 factors that constitute the shadow of the OPVR value from four aspects, as determined by expert consultation specifically, the options of the questionnaire are categorized into five levels, with the following ratings: 1 indicates negligible influence, 2 indicates small influence, 3 indicates moderate influence, 4 indicates large influence, and 5 indicates substantial influence. Based on your previous experience, please ascertain the extent to which the following influences affect the OPVR value; all questions are single-choice.

**S4 Table Questionnaire on the extent to which factors influence the OPVR value**

| Factors affecting the formation of the OPVR value | | Degree of impact | | | | |
| --- | --- | --- | --- | --- | --- | --- |
|  |  | 1 | 2 | 3 | 4 | 5 |
| Variety value | Consumer Demand |  |  |  |  |  |
|  | Variety Quality |  |  |  |  |  |
|  | Market Size |  |  |  |  |  |
| Technical level | Technical Barrier |  |  |  |  |  |
|  | Technology Maturity |  |  |  |  |  |
|  | Production Cost |  |  |  |  |  |
| Marketing and brand | Flower Language Imagery |  |  |  |  |  |
|  | Marketing Platform |  |  |  |  |  |
|  | Logistics System |  |  |  |  |  |
| Intellectual Property Protection | Protection Policy |  |  |  |  |  |
|  | Law Enforcement |  |  |  |  |  |
|  | Public Awareness |  |  |  |  |  |
