# Supplementary material for: The mechanism of the ornamental plant variety rights value formation and enhancement strategy based on SEM-SD
Source: PLoS One. 2025 Dec 19;20(12):e0336934. doi: 10.1371/journal.pone.0336934 (PMC12716751; doi:10.1371/journal.pone.0336934)
Supplement: S1 File. — S1 Table. Questionnaire on Factors Affecting the Formation of the Value of the Ornamental Plant Variety Rights. S2 Table. Expert structure. S3 Table. Mention degree of influencing factors. S4 Table. Questionnaire on the extent to which factors influence the OPVR value. S5 Table. Fundamental Statistical Information of Survey Questionnaire. S6 Table. Descriptive Statistics of Sample Data Variables (N = 220). S7 Table. Reliability test of the survey questionnaire. S8 Table. KMO and Bartlett’s test for overall questionnaire data. S9 Table. KMO and Bartlett’s test for each variable in the questionnaire data. S10 Table. Factor Rotation Component Matrix. S11 Table. Adaptability Test for the Second Order Confirmatory Factor Analysis of the OPVR value. S12 Table. Initial hypothesis model suitability test. S13 Table. Fitting of the Intrinsic Structure of the Initial Hypothesis Model. S14 Table. Hypothesis model fitness test following the first revision. S15 Table. Path coefficients of the hypothesis model and the results of their significance test results following one modification. S16 Table. Details of System State Variables. S17 Table. Details of Rate Variables in the Value System of the OPVR. S18 Table. List of Auxiliary Variables in the Value System of the OPVR. S19 Table. Simulation comparison of variety kernel modules. S20 Table. Simulation comparison of variety kernel modules. S21 Table. Simulation comparison of marketing module. S22 Table. Simulation comparison of intellectual property protection sales module. S1 Fig. Prediction of the OPVR value. (ZIP) [file pone.0336934.s001.zip › supporting information/S5 Table.docx]

**S5 Table Fundamental Statistical Information of Survey Questionnaire**

| Work unit | Years of experience | Frequency | Percentage |
| --- | --- | --- | --- |
| seed business | 0-5 years | 52 | 23.64% |
| seed business | 6-10 years | 68 | 30.91% |
| seed business | 11-15 years | 35 | 15.91% |
| seed business | More than 15 years | 26 | 11.82% |
| universities and colleges | 0-5 years | 16 | 7.27% |
| universities and colleges | 6-10 years | 12 | 5.45% |
| universities and colleges | 11-15 years | 8 | 3.64% |
| universities and colleges | More than 15 years | 3 | 1.36% |
